# Detecting Differentially Expressed Genes with RNA-seq Data Using Backward Selection to Account for the Effects of Relevant Covariates

**DOI:** 10.1007/s13253-015-0226-1

**Published:** 2015-10-01

**Authors:** Yet Nguyen, Dan Nettleton, Haibo Liu, Christopher K. Tuggle

**Affiliations:** Department of Statistics, Iowa State University, Ames, IA 50010 USA; Department of Animal Science, Iowa State University, Ames, IA 50010 USA; Institute of Mathematics, VAST, Hanoi, Vietnam

**Keywords:** False discovery rate, Generalized linear model, Quasi-likelihood, Residual feed intake

## Abstract

A common challenge in analysis of transcriptomic data is to identify differentially expressed genes, i.e., genes whose mean transcript abundance levels differ across the levels of a factor of scientific interest. Transcript abundance levels can be measured simultaneously for thousands of genes in multiple biological samples using RNA sequencing (RNA-seq) technology. Part of the variation in RNA-seq measures of transcript abundance may be associated with variation in continuous and/or categorical covariates measured for each experimental unit or RNA sample. Ignoring relevant covariates or modeling the effects of irrelevant covariates can be detrimental to identifying differentially expressed genes. We propose a backward selection strategy for selecting a set of covariates whose effects are accounted for when searching for differentially expressed genes. We illustrate our approach through the analysis of an RNA-seq study intended to identify genes differentially expressed between two lines of pigs divergently selected for residual feed intake. We use simulation to show the advantages of our backward selection procedure over alternative strategies that either ignore or adjust for all measured covariates.

## Introduction

A standard challenge in transcriptomic data analysis is to identify genes whose mean transcript abundance levels differ across the levels of a categorical factor of primary scientific interest (e.g., treatment, genotype, tissue, or disease state). Such genes are typically referred to as differentially expressed (DE). Currently, the leading technology used to detect DE genes is RNA sequencing (RNA-seq). In raw form, RNA-seq data contain information about the identity of bases in short RNA sequence fragments known as reads. For the purpose of identifying DE genes, the number of reads matching each of thousands of gene sequences is determined for each of several experimental or observational units. These read counts serve as measures of RNA abundance. Typically, a generalized linear model with a log link and a negative binomial response is fit to the count data for each gene, and DE genes are identified by testing, for each gene, whether a model parameter or linear combination of model parameters is zero.

RNA-seq datasets often contain several covariates in addition to the factor of primary scientific interest. As in any experiment or observational study, covariates may hold information about heterogeneity of the experimental or observational units used in the investigation. Other covariates in an RNA-seq dataset may track variation that is created during the complex process of measuring RNA transcript abundance levels using RNA-seq technology. If covariates are ignored when searching for DE genes, the unaccounted for variation in expression levels associated with variation in covariates may obscure the association of expression levels with the primary factor of interest. On the other hand, explicitly accounting for the effects of all covariates in data analysis may be inefficient when some covariates are actually unassociated or only weakly associated with expression levels. Either ignoring relevant covariates or accounting for the effects of irrelevant covariates reduces power for identifying DE genes. Unfortunately, the power problem is exacerbated by the low sample sizes common in expensive RNA-seq experiments.

To address the challenge of identifying DE genes with RNA-seq datasets that include covariates, we propose a backward selection algorithm for selecting a subset of covariates whose effects are estimated and adjusted for when testing for differential expression. Our goal is to find one subset of all available covariates to include in every gene-specific generalized linear model. Although it is possible (and perhaps most likely) that the subset of covariates relevant for one gene is different than the subset of covariates relevant for another, we seek one subset of covariates common to all genes for two main reasons. First, the number of experimental/observational units is often relatively small in RNA-seq datasets, especially in agricultural applications. Small sample sizes lead to unreliable model selection and considerable uncertainty in models selected separately for tens of thousands of genes. Second, for purposes of interpretability, it is useful to test for differential expression by adjusting for the same set of covariates for all genes. Identifying DE genes involves testing whether one (or more) partial regression coefficients in a generalized linear model are zero. If different covariates are used for different genes, the interpretation of partial regression coefficients—and consequently the definition of differential expression—changes from gene to gene. A shifting definition of what it means for a gene to be DE is undesirable when reporting results. Instead, we choose one subset of covariates for all genes and attempt to answer the following question: If we adjust for the effects of the subset of variables that tends to be most relevant when considering all genes, do we see significant differences in mean transcript abundance levels across the levels of the factor of primary scientific interest?

As a motivating example, we consider RNA-seq measures of transcript abundance in blood samples from 31 pigs of two genetic lines created by selection on the basis of residual feed intake (RFI). RFI is computed as the observed feed intake of an animal minus an estimate of the feed intake that would be expected considering that animal’s growth characteristics. Pigs from the high residual feed intake (HRFI) line tend to eat more feed than expected adjusted for their growth, while pigs from the low residual feed intake (LRFI) line tend to eat less than expected adjusted for their growth. Because feed is the largest single cost incurred by US pork producers, pigs of the LRFI line have economically desirable feeding and growth characteristics, and understanding the transcriptional differences between these lines is of scientific interest.

Finding genes differentially expressed between lines is complicated by heterogeneity among pigs, heterogeneity among the blood samples extracted from pigs, and heterogeneity among the processed and measured RNA samples derived from the blood samples. A total of 13 categorical and continuous covariates (described in detail in the Appendix) are available for tracking and accounting for this heterogeneity. The backward selection procedure that we formally define in Sect. 2.3 starts by fitting, for each gene, a full generalized linear model with a negative binomial response and a log link that includes the effects of primary interest due to line as well as effects for all 13 covariates. Using criteria described in Sect. 2.4, the least relevant variable when considering results from all genes is dropped, and the resulting reduced model is fit for all genes. This process continues until the variable identified as least relevant is the factor of scientific interest (line in our example). This backward selection procedure produces a sequence of increasingly smaller subsets of covariates, starting with all covariates and progressing, one removed variable at a time, down to a subset of covariates most strongly associated with transcript abundance levels when considering the results for all genes. From this sequence of subsets of covariates, we determine the subset of covariates that, when accounted for, leads to identification of the greatest number of genes differentially expressed across the levels of the factor of primary scientific interest (i.e., line).

The mechanics of our backward selection procedure are similar to those of the usual backward selection procedure used in multiple regressions in that the variable least significant (by some criterion) is removed at each step. One major difference between our proposed procedure and the usual backward selection procedure for multiple regressions is that we are dealing simultaneously with thousands of response variables rather than a single response. A second major difference (related to the first) is that the subset of variables we ultimately select from the sequence of subsets generated by backward selection is determined by maximizing the number of rejected null hypotheses for a test of interest across thousands of response variables. This strategy is motivated by the knowledge that both including irrelevant covariates and excluding relevant covariates can act to reduce power. Thus, selecting the set of covariates that maximizes the number of rejected hypotheses for the test of interest is a natural strategy for identifying the most relevant covariates.

In a simulation study presented in Sect. 4, we show that our backward selection procedure is effective at selecting the truly relevant covariates when the truly relevant covariates are the same for all genes. In this idealized situation, our simulations also show that the false discovery rate (FDR) can be controlled when tests for differential expression are conducted while adjusting for the effects of the covariates selected using our backward selection procedure. We also show that FDR can still be controlled even when the set of truly relevant covariates differs across genes. However, results must be carefully interpreted if some excluded covariates are associated with the factor of primary scientific interest.

Prior to presenting our differential expression analysis of the RFI RNA-Seq dataset in Sect. 3, we provide more details about generalized linear models and significance testing for RNA-seq read count data in Sects. 2.1 and 2.2. We formally define our proposed backward selection procedure in Sect. 2.3. Section 2.4 covers two measures of covariate relevance that can be used to choose covariates for removal in each step of backward selection. We compare the performance of the backward selection algorithm with alternative methods in a simulation study presented in Sect. 4. The paper concludes with a discussion in Sect. 5.

## Methods

### Generalized Linear Models for RNA-seq Read Count Data

Consider the analysis of *m* genes using RNA-seq read count data from *n* experimental or observational units. For $g=1,\ldots , m$ and $i=1,\ldots , n$, let $y_{gi}$ be the read count for gene *g* from experimental/observational unit *i*. Let $\varvec{x}_i=(\varvec{x}'_{i1},\ldots , \varvec{x}'_{ik})'$ denote a vector of known explanatory variable values for the *i*th unit. The *k* explanatory variables include the factor of primary scientific interest and $k-1$ continuous or multi-level categorical covariates. Without loss of generality, we assume that $\varvec{x}_{i1}$ (the leading subvector of $\varvec{x}_i$) is a vector of zero-one indicator variable values that code for the level of the factor of primary scientific interest associated with unit *i*. The number of components of $\varvec{x}_{i1}$ is one less than the number of levels of the factor of primary scientific interest. For example, for the RFI dataset discussed in Sect. 1 and in more detail in Sect. 3 and the Appendix, $\varvec{x}_{i1}$ is simply a single indicator variable that takes the value 1 if the *i*th pig is from the LRFI line and the value 0 if the *i*th pig is from the HRFI line. Each of the other vectors $\varvec{x}_{i2}, \ldots , \varvec{x}_{ik}$ corresponds to either a continuous or categorical covariate that is not of primary scientific interest. Vectors for continuous covariates have only one element while vectors corresponding to categorical covariates consist of indicator variable values with one less indicator than the number of levels of the categorical covariate. Finally, let $o_i$ be the normalization offset computed for unit *i*. The normalization offsets account for differences in the thoroughness of sequencing across the units. A variety of normalization offsets have been proposed in the literature (see, e.g., Marioni et al. 2008; Mortazavi et al. 2008; Robinson and Oshlack 2010; Anders and Huber 2010; Bullard et al. 2010; Risso et al. 2014a, b, and references therein). Throughout this paper, we set $o_i$ to be the log of the 0.75 quantile of unit *i* read counts in accordance with the recommendation of Bullard et al. (2010).

As is popular in RNA-seq data analysis, we use, as a working assumption, that the read counts for gene *g* ($y_{g1},\ldots , y_{gn}$) are independent and that $y_{gi} \sim \text {NB}(\mu _{gi}, \omega _g)$, where $\text {NB}(\mu _{gi}, \omega _g)$ is the negative binomial distribution with mean $\mu _{gi}$, dispersion parameter $\omega _g$, and variance $\mu _{gi} + \omega _g \mu _{gi}^2$. Letting ${\mathcal {S}}$ represent a subset of $\{1,\ldots ,k\}$ that contains 1, we consider log-linear models of the form

1 $$\begin{aligned} \log (\mu _{gi}) = o_{i} + \beta _{g0|{\mathcal {S}}} + \sum _{j\in {\mathcal {S}}} \varvec{x}_{ij}'\varvec{\beta }_{gj|{\mathcal {S}}}, \end{aligned}$$

where $\beta _{g0|{\mathcal {S}}}$ is an unknown intercept parameter, $\varvec{\beta }_{g1|{\mathcal {S}}}$ is an unknown parameter vector for the factor of primary scientific interest, and $\varvec{\beta }_{gj|{\mathcal {S}}}$ is a vector of unknown covariate effects for each $j\in {\mathcal {S}}\setminus \{1\}$. The set ${\mathcal {S}}$ is included in the parameter subscripts to emphasize that the meaning of partial regression coefficients depends on all the covariates included in the model. We use ${\mathcal {S}}^*$ to represent the unknown set containing 1 and the largest subset of $\{2,\ldots , k\}$ such that $j\in {\mathcal {S}}^*\setminus \{1\}$ implies $\varvec{\beta }_{gj|{\mathcal {S}}} \ne \varvec{0}$ for some $g \in \{1,\ldots , m\}$. This makes ${\mathcal {S}}^*\setminus \{1\}$ the set of all indices corresponding to covariates relevant for at least one gene.

For all $g=1,\ldots , m$, we wish to test $H_{0g1}^{{\mathcal {S}}^*} : \varvec{\beta }_{g1|{\mathcal {S}}^*}=\varvec{0}$. If $H_{0g1}^{{\mathcal {S}}^*}$ is false, gene *g* is said to be differentially expressed (DE). Otherwise, gene *g* is said to be equivalently expressed (EE). Because the set of all relevant covariates ${\mathcal {S}}^*\setminus \{1\}$ is unknown, we cannot directly test $H_{0g1}^{{\mathcal {S}}^*}$ for any gene *g*. Instead, we use a backward selection procedure to first identify a set of covariates $\hat{\mathcal {S}}^*$ to approximate ${\mathcal {S}}^*$. Then, for each gene *g*, we fit the (possibly misspecified) model in which the true equation defining $\log (\mu _{gi})$ in (1) is replaced by

2 $$\begin{aligned} \log (\mu _{gi}) = o_{i} + {\beta }_{g0|\hat{\mathcal {S}}^*} + \sum _{j\in \hat{\mathcal {S}}^*} \varvec{x}_{ij}'{\varvec{\beta }}_{gj|\hat{\mathcal {S}}^*}. \end{aligned}$$

Note that the regression coefficients in (2) are the same as the partial regression coefficients in (1) whenever $\hat{\mathcal {S}}^*={\mathcal {S}}^*$. Even if $\hat{\mathcal {S}}^*\ne {\mathcal {S}}^*$, the partial regression coefficients of interest given by ${\varvec{\beta }}_{g1|{\mathcal {S}}^*}$ may be similar to ${\varvec{\beta }}_{g1|\hat{\mathcal {S}}^*}$ if $\hat{\mathcal {S}}^*$ includes the most relevant covariates. In such situations, reasonable decisions about whether ${\varvec{\beta }}_{g1|{\mathcal {S}}^*}=\varvec{0}$ may be reached by testing $H_{0g1}^{\hat{\mathcal {S}}^*} : {\varvec{\beta }}_{g1|\hat{\mathcal {S}}^*}=\varvec{0}$. Thus, for each $g\in \{1,\ldots , m\}$, we test $H_{0g1}^{\hat{\mathcal {S}}^*} : {\varvec{\beta }}_{g1|\hat{\mathcal {S}}^*}=\varvec{0}$, and we use the *p*-values from these *m* tests to declare a subset of the *m* genes to be differentially expressed.

### Significance Testing for RNA-seq Read Count Data

A variety of methods have been proposed for testing the significance of regression coefficients in generalized linear models for RNA-seq read count data. Some prominent examples include Lu et al. (2005), Robinson and Smyth (2008a, b), Anders and Huber (2010), Hardcastle and Kelly (2010), Di et al. (2011), Van De Wiel et al. (2012), and McCarthy et al. (2012). A recent review of methods was provided by Lorenz et al. (2014). To conduct our tests for differential expression and to assess the significance of covariates, we use the R (R Core Team 2012) QuasiSeq package, which implements the quasi-likelihood testing method developed by Lund et al. (2012). This approach was recently found by Burden et al. (2014) to be the “best performing package in the sense that it achieves a low FDR which is accurately estimated over the full range of *p*-values.”

In brief, the QuasiSeq method uses a hierarchical model for gene-specific quasi-dispersion parameters to obtain quasi-dispersion parameter estimates that are stabilized by borrowing information across genes. For each gene, the usual likelihood ratio test statistic for testing the significance of a subvector of regression coefficients is then scaled by the inverse of the estimated quasi-dispersion parameter. This scaled test statistic is then compared to an appropriate central *F* distribution to obtain an approximate *p*-value. Approximate control of the false discovery rate (FDR) at any desired level $\alpha$ is obtained by converting the *p*-values to *q*-values (Storey 2002) and rejecting a null hypothesis if and only if its corresponding *q*-value is less than $\alpha$. When computing *q*-values by the method of Storey (2002), an estimate of $m_0$, the number of true null hypotheses among all *m* null hypotheses tested, is required. We use the histogram-based method of Nettleton et al. (2006) to estimate $m_0$. Desirable theoretical characteristics of a closely related histogram-based approach were demonstrated by Liang and Nettleton (2012).

The denominator degrees of freedom parameter for the *F* distribution used to obtain *p*-values in the quasi-likelihood analysis are bounded below by the sample size minus the number of estimated partial regression coefficients and, all else equal, will decrease as irrelevant covariates are included in the model. Decreased denominator degrees of freedom can result in a loss in power for detecting DE genes. On the other hand, excluding relevant covariates will increase the denominator degrees of freedom at the cost of larger quasi-dispersion parameter estimates due to lack of model fit. Because the estimated quasi-dispersion parameters are the denominators of the *F* statistics, larger quasi-dispersion parameter estimates lead to smaller *F* statistics and, again, reduced power for identifying differentially expressed genes. For these reasons, finding the most relevant set of covariates is crucial for differential expression analysis.

### The Proposed Backward Selection Algorithm

Let ${\mathcal {S}}$ be any subset of $\{1,\ldots ,k\}$. For any $j\in {\mathcal {S}}$, let $\varvec{p}_{j|{\mathcal {S}}}$ denote the vector of $m\,p$-values obtained by testing $H_{0gj}^{{\mathcal {S}}}:\varvec{\beta }_{gj|{\mathcal {S}}}=\varvec{0}$ for each gene $g=1,\ldots , m$. Let $r(\varvec{p}_{j|{\mathcal {S}}})$ be a measure of the relevance of $\varvec{x}_j$ in model (1); as an example, the simplest of the two relevance measures we consider in this paper (see Sect. 2.4) is the number of elements of $\varvec{p}_{j|{\mathcal {S}}}$ less than 0.05. Let ${\mathcal {S}}_1=\{1,\ldots ,k\}$ and consider an iterative procedure whose $\ell \hbox {th}$ iteration is as follows:

1. Compute $\varvec{p}_{j|{\mathcal {S}}_{\ell }}$ for all $j \in {\mathcal {S}}_\ell$.
2. Let $\varvec{q}_\ell$ be the vector of *q*-values obtained from $\varvec{p}_{1|{\mathcal {S}}_{\ell }}$.
3. Let $R_\ell (\alpha )$ be the number of *q*-values in $\varvec{q}_\ell$ less than or equal to a user-defined FDR threshold $\alpha$.
4. Find $j^*$ so that $r(\varvec{p}_{j^*|{\mathcal {S}}})\le r(\varvec{p}_{j|{\mathcal {S}}})$ for all $j \in {\mathcal {S}}_\ell$.
5. If $j^*=1$, stop iterating. Otherwise, carry out the $\ell +1\hbox {st}$ iteration with ${\mathcal {S}}_{\ell +1}={\mathcal {S}}_\ell \setminus \{j^*\}$.

Suppose the iterative procedure concludes after *L* iterations, and let $\ell ^*$ be the smallest element of $\{1,\ldots ,L\}$ such that $R_{\ell ^*}(\alpha )\ge R_\ell (\alpha )$ for all $\ell \in \{1,\ldots ,L\}$. We set $\hat{{\mathcal {S}}}^*={\mathcal {S}}_{\ell ^*}$ and base our inference about differential expression on the fit of model (2). By the definition of $\ell ^*$, this analysis will maximize the number of genes declared to be differentially expressed (at FDR threshold $\alpha$) over the *L* models that correspond to the *L* explanatory variable index sets ${\mathcal {S}}_1 \supset \cdots \supset {\mathcal {S}}_L$. Despite maximizing the number of genes declared differentially expressed over the sequence of models, we show through simulation studies in Sect. 4 that this approach can control the false discovery rate at desired levels.

### Measures of Variable Relevance

For a given ${\mathcal {S}}\subseteq \{1,\ldots , k\}$ and any $j\in {\mathcal {S}}$, we consider variable *j* to be an irrelevant variable if

3 $$\begin{aligned} H_{0gj}^{{\mathcal {S}}}:\varvec{\beta }_{gj|{\mathcal {S}}}=\varvec{0}\text { is true for all } g=1,\ldots ,m. \end{aligned}$$

When (3) holds, each element of $\varvec{p}_{j|{\mathcal {S}}}$ will be uniformly distributed on (0, 1) whenever the test used to produce the elements of $\varvec{p}_{j|{\mathcal {S}}}$ has size equal to the significance level for all significance levels in (0, 1). If the test used to produce the elements of $\varvec{p}_{j|{\mathcal {S}}}$ is unbiased for all significance levels, then an element of $\varvec{p}_{j|{\mathcal {S}}}$ corresponding to a false null hypothesis will have a distribution stochastically smaller than $\mathrm{uniform}(0,1)$ and a density that is decreasing on the interval (0, 1). Based on this reasoning, the empirical distribution of the elements of $\varvec{p}_{j|{\mathcal {S}}}$ provides information about the relevance of variable *j* in the model that includes the explanatory variables whose indices are contained in ${\mathcal {S}}$. An empirical distribution close to uniform or stochastically larger than uniform implies little relevance while an empirical distribution with a clear excess of small *p*-values relative to a uniform distribution implies relevance of variable *j* for at least least some appreciable number of genes.

**Fig. 1:**
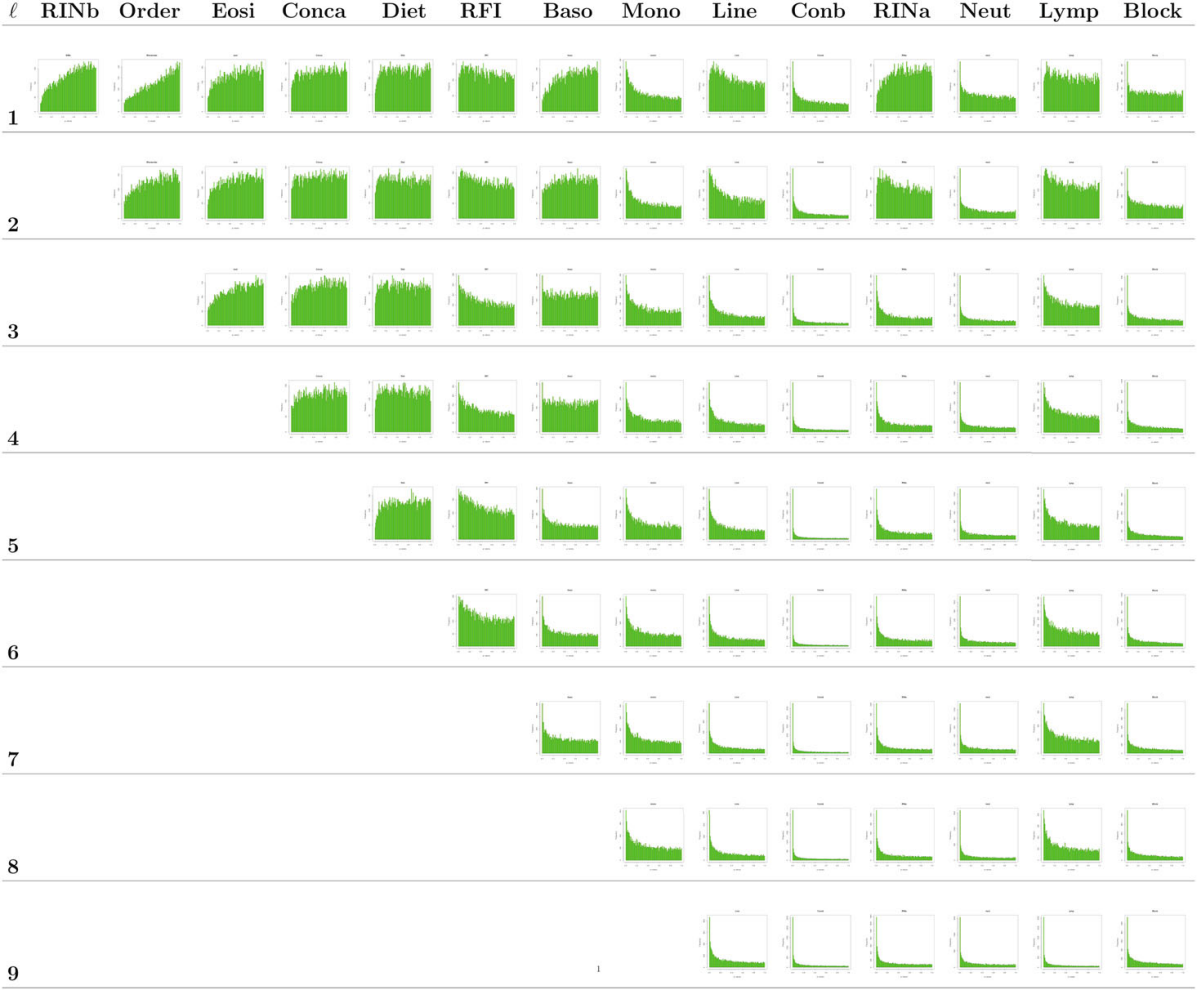
Histograms of *p*-values at each iteration of the backward selection procedure applied to the RFI RNA-seq dataset using the number of *p*-values less than 0.05 (*p*.05) as the measure of covariate relevance. Rather than using a common upper limit for each histogram’s vertical axis, the upper limit varies across histograms to accommodate the height of the tallest bar in each histogram. Using variable upper limits makes it easier to see differences between the histogram shapes of relevant and irrelevant covariates.

In practice, the tests used to assess significance are only approximate, each observed *p*-value is only a single draw from its marginal distribution, and dependence among genes leads to dependence among *p*-values. For all of these reasons, empirical distributions composed of one *p*-value from each gene can have shapes that are neither uniform nor stochastically smaller than uniform. Nonetheless, measuring the extent to which an empirical distribution of the elements of $\varvec{p}_{j|{\mathcal {S}}}$ departs from uniform toward a distribution with a decreasing density on (0, 1) can provide a useful measure of relevance for variable *j*. As an example, the histograms in the first row of Fig. 1 show the empirical distribution of the elements of $\varvec{p}_{j|{\mathcal {S}}}$ for each $j \in {\mathcal {S}}=\{1,\ldots ,14\}$. Based on visual inspection, covariates like *RINb*, *Conca*, *Order*, *Diet*, and *Eosi* appear irrelevant in the full model, while covariates like *Concb*, *Neut*, *Mono*, and *Block* appear relevant.

There are many ways to formally measure the relevance of explanatory variable *j* through definition of a relevance function $r(\cdot )$ that maps $\varvec{p}_{j|{\mathcal {S}}}$ to the real line. We consider two choices for $r(\cdot )$, one relatively simple and one more complicated. It turns out that both measures of relevance lead to similar performance for our backward selection and testing procedure. As noted in the previous section, the simpler of our two relevance measure sets $r(\varvec{p}_{j|{\mathcal {S}}})$ to the number of elements of $\varvec{p}_{j|{\mathcal {S}}}$ less than 0.05. We use *p*.05 as an abbreviation for this criterion in the remainder of the paper. The more complicated version of $r(\cdot )$ is described as follows.

Given a vector of *p*-values $\varvec{p}=(p_1, \ldots , p_m)'$, let $\hat{F}_m(\cdot )$ be the empirical distribution function of the elements of $\varvec{p}$. If we were to assume that the elements of $\varvec{p}$ were an independent and identically distributed sample from a distribution with cumulative distribution function $F(\cdot )$, then $\hat{F}_m(\cdot )$ is known to be the nonparametric maximum likelihood estimator of $F(\cdot )$. If we were to assume that the distribution defined by $F(\cdot )$ has a non-increasing density, then the nonparametric maximum likelihood estimator of $F(\cdot )$, subject to the constraint of a non-increasing density, is given by $\tilde{F}_m(\cdot )$, the least concave majorant of $\hat{F}_m(\cdot )$ (Grenander 1956). If we let

4 $$\begin{aligned} r(\varvec{p})=\sqrt{m}\sup _{x \in (0,1)} [\tilde{F}_m(x)-x], \end{aligned}$$

then $r(\varvec{p})$ is a Kolmogorov–Smirnov-type statistic that measures the extent to which the empirical distribution of the elements of $\varvec{p}$ departs from a uniform(0, 1) distribution toward a distribution with a decreasing density on (0, 1). Henceforth, we refer to this measure of variable relevance as the GKS criterion (short for Grenander–Kolmogorov–Smirnov).

## Analysis of the RFI RNA-Seq Dataset

The proposed backward selection algorithm was used to analyze the RFI RNA-seq dataset introduced in Sect. 1 and described in more detail in the Appendix. Recall that the primary scientific goal is to identify genes whose mean transcript abundance levels, adjusted for relevant covariates, differ between the LRFI and HRFI lines. The dataset consists of read counts for 12,280 genes for each of 31 pigs. As is customary in RNA-seq analysis, this dataset excludes genes with predominantly low read counts because genes with low read counts contain little information about differential expression and can lead to computation problems when attempting to find maximum likelihood estimates of partial regression coefficients in our negative binomial generalized linear models. Thus, the 12,280 genes analyzed in this study each have average read counts of at least 8 and no more than 27 zero counts across the 31 pigs. This same threshold for gene inclusion was used throughout the simulation study described in Sect. 4.

Table 1 and Fig. 1 summarize the results of the backward selection algorithm when *p*.05 is used as the measure of covariate relevance. The covariate *RINb* was the first to be removed from the full model, followed in subsequent iterations by the covariates *Order*, *Eosi*, *Conca*, *Diet*, *RFI*, *Baso*, and *Mono*. At the 9th iteration, *Line* was judged to be the least relevant factor, and thus, the backward selection procedure terminated.

Figure 1 shows that several of the variables have *p*-value distributions that change substantially as covariates are removed during backward selection. The *p*-value distribution of *Lymp*, for example, is close to uniform in the full model but gradually concentrates increasing mass on small *p*-values as variables are excluded from the full model. *Lymp* is a continuous covariate measured for each blood sample and is not orthogonal to any variable in the dataset. *Lymp* has particularly high Pearson correlation coefficients (0.63, 0.67, and 0.85) with three other blood sample variables, *Eosi*, *Baso*, and *Mono*. Due to this non-orthogonality, it is not surprising to see changes in the significance of the *Lymp* partial regression coefficients as *Eosi*, *Baso*, and *Mono* are eliminated from the model after iterations 3, 7, and 8, respectively. Many other changes in *p*-value histograms from iteration to iteration can be explained similarly.

As the bottom row of Table 1 shows, the model corresponding to iteration $\ell =7$ yielded the greatest number ($R_7(0.05) = 448$) of *q*-values no larger than 0.05 for the *Line* test. Hence, the backward selection procedure resulted in the declaration of 448 genes as differentially expressed between the LRFI and HRFI lines while controlling for the effects of the covariates *Baso*, *Mono*, *Concb*, *RINa*, *Neut*, *Lymp*, and *Block*. For this dataset, the backward selection procedure using the GKS criterion to measure covariate relevance deleted variables in a slightly different order but selected the same final model and, therefore, provided results identical to backward selection using *p*.05 to measure covariate relevance.

**Table 1:** The first 14 rows show the number of *p*-values less than 0.05 for each covariate at each iteration of the backward selection algorithm applied to the RFI RNA-seq data.

	$\ell =1$	$\ell =2$	$\ell =3$	$\ell =4$	$\ell =5$	$\ell =6$	$\ell =7$	$\ell =8$	$\ell =9$
RINb	202								
Order	235	324							
Eosi	303	340	320						
Conca	450	503	421	409					
Diet	392	497	489	507	335				
RFI	585	708	1004	1042	879	917			
Baso	255	458	742	742	1262	1396	1275		
Mono	1400	1531	1293	1519	1371	1496	1506	1432	
Line	722	1221	1682	1793	1676	1949	2303	2139	2235
Concb	1680	2635	3015	3326	4353	4414	4385	4331	4153
RINa	281	681	1939	2020	2100	2118	2543	2594	2997
Neut	1138	1704	2123	2155	2290	2350	2352	2987	2919
Lymp	625	818	1119	1251	1350	1393	1354	1606	4225
Block	967	1259	1867	2152	2379	2456	2406	2380	2440
$R_\ell (0.05)$	2	2	1	1	0	46	448	337	379

The last row $R_{\ell }(0.05)$ is the number of *q*-values less than or equal to 0.05 for the test of the *Line* factor in each iteration

The results of our proposed backward selection procedure can be contrasted with two simple alternative strategies that might be used in practice. The first such strategy is to account for all covariates regardless of whether the data suggest they are relevant. As shown in the first column and last row of Table 1, fitting the full model yielded only two genes with *q*-values less than 0.05 for the *Line* test. The second strategy is to ignore all covariates. This is the only strategy available to researchers who do not measure or record covariates, and it might be the most commonly used strategy, considering that many published RNA-seq studies of differential expression do not mention covariates. When the 13 covariates in the RFI RNA-seq analysis were ignored, 251 genes had *q*-values less than or equal to 0.05 for the *Line* test. Both of these alternative strategies identified far fewer differentially expressed genes than our proposed backward selection procedure. Via simulation, we evaluate the efficacy of these simple strategies relative to our backward selection procedure in the next section.

## Simulation Study

We considered three simulation scenarios described in detail in Sects. 4.1, 4.2, and 4.3, respectively. We compared analysis approaches with respect to their ability to identify differentially expressed genes while controlling FDR. Such comparisons require simulated datasets to contain both EE and DE genes. Within each scenario, we varied $\pi _0=$ the proportion of EE genes over the values 0.6, 0.7, 0.8, and 0.9. Within each scenario and for each value of $\pi _0 \in \{0.6, 0.7, 0.8, 0.9\}$, we simulated 100 datasets. Each dataset included read counts for 31 pigs and 5000 genes simulated from independent negative binomial distributions. The log of each negative binomial mean was set to be a linear combination of covariates as in Eq. (1), with ${\mathcal {S}}$ specifically defined in each scenario. Except where otherwise noted in Sect. 4.3, covariates for the 31 pigs were held fixed at the values observed for the actual RFI data. The true values of partial regression coefficients and negative binomial dispersion parameters were set based on values estimated from the RFI data, and EE genes were established by setting to zero the partial regression coefficient for the *Line* indicator variable as detailed in the following sections.

### Simulation Scenario 1: Same Set of Relevant Covariates for Every Gene

The first simulation scenario provides a favorable case for our backward selection procedure in which the same set of covariates is relevant for every gene. As the common set of relevant covariates, we used those identified by our backward selection procedure when applied to the RFI dataset in Sect. 3, i.e., *Line*, *Baso*, *Lymp*, *Mono*, *Neut*, *Concb*, *RINa*, and *Block*. As true parameter values for simulating new data, we used the dispersion parameter estimates and the partial regression coefficient estimates from the fit of the selected model to the RFI data, except that we set partial regression coefficients on the *Line* indicator variable to zero for a subset of genes to permit simulation of EE genes. More specifically, the $\hat{m}_0=7795$ least significant partial regression coefficients for *Line* were set to zero, where $\hat{m}_0=7795$ is the estimated number of *Line* partial regression coefficients equal to zero when the method of Nettleton et al. (2006) is applied to *Linep*-values from the fit of the selected model to the RFI data. This strategy yielded a parameter vector (consisting of a dispersion parameter and partial regression coefficients) for each of 7795 EE genes and each of $12280-7795=4485$ DE genes. To simulate any particular dataset for a given value of $\pi _0\in \{0.6,0.7,0.8,0.9\}$, we randomly sampled $5000 \cdot \pi _0$ EE gene parameter vectors and $5000 \cdot (1-\pi _0)$ DE gene parameter vectors. The selected parameter vectors and observed covariates for the 31 pigs were used to simulate a $5000 \times 31$ dataset of negative binomial read counts. Random selection of parameters and generation of data were independently repeated 100 times to obtain the 100 datasets for each value of $\pi _0\in \{0.6,0.7,0.8,0.9\}$ as described in the introduction to Sect. 4.

### Simulation Scenario 2: Different Sets of Relevant Covariates for Different Genes

The second simulation scenario is designed to evaluate our backward selection procedure when, contrary to our working assumption, different sets of covariates are relevant for different genes within each dataset. In simulation scenario 2, each dataset was simulated using exactly the same procedure described in Sect. 4.1, except that instead of generating data for all 5000 genes using one set of relevant covariates, data for 1250 genes were simulated from each of four covariate sets. The covariate sets we considered are sets ${\mathcal {S}}_6,\, {\mathcal {S}}_7,\, {\mathcal {S}}_8$, and ${\mathcal {S}}_9$, which correspond to iterations $\ell =6,7,8$ and 9 form the RFI data analysis is Sect. 3. The largest of these covariate sets (${\mathcal {S}}_6$) contains the covariate *RFI* in addition to the covariates considered in Sect. 4.1 (those in ${\mathcal {S}}_7$). Covariate sets ${\mathcal {S}}_8$ and ${\mathcal {S}}_9$ differ from ${\mathcal {S}}_7$ by the exclusion of covariates *Baso* and both *Baso* and *Mono*, respectively.

### Simulation Scenario 3: Orthogonal Covariates

As described in the Appendix, the covariate *RFI* provides a continuous measure of residual feed intake for each of the 31 pigs in the study. Because the LRFI and HRFI lines were created by selecting on residual feed intake for several generations, it is not surprising that the LRFI pigs in our study tend to have lower *RFI* values than the HRFI pigs in our study. Thus, the *RFI* covariate is strongly associated with the factor *Line* in our dataset. This association makes it difficult to distinguish the direct effects of *Line* from the direct effects of *RFI* on transcript abundance levels. To remove this partial confounding in the third simulation scenario, the average *RFI* value for pigs from the LRFI line was subtracted from each LRFI pig’s *RFI* value. Likewise, the average *RFI* value for pigs from the HRFI line was subtracted from each HRFI pig’s *RFI* value. After these subtractions, the altered *RFI* values sum to zero within each line so that the altered *RFI* variable is orthogonal to the *Line* factor. The simulation strategy described in Sect. 4.2 was then repeated with the altered *RFI* values in place of the original *RFI* values.

### Simulation Results

We analyzed the simulated datasets using model (2) with five different strategies for choosing $\hat{\mathcal {S}}^*$: all available covariates (Full), only the factor of primary interest (Line Only), the backward selection procedure with the *p*.05 measure of covariate relevance (Backward), the backward selection procedure with the GKS measure of covariate relevance, and $\hat{\mathcal {S}}^*={\mathcal {S}}^*$, i.e., using the true set of covariates that was actually used to simulate the data for each gene (Oracle). Of course, the Oracle procedure cannot be used in practice, but its inclusion provides a useful reference measure of the performance achieved if covariate selection were perfect.

For all five analysis strategies, the QuasiSeqR package was used to compute a *p*-value for testing the significance of the partial regression coefficient on the *Line* indicator variable for each gene. These *p*-values were converted to *q*-values (as described in Sect. 2.2), and genes with *q*-values no larger than 0.05 were declared DE. We evaluated each procedure’s performance according to three criteria: the incurred FDR when FDR is nominally controlled at 5%, the number of true positive (NTP) declarations of differential expression, and the partial area under the receiver operating characteristic curve (PAUC) corresponding to false positive rates less than or equal to 0.05. These performance criteria assess error control, power, and the ability to distinguish EE and DE genes from one another, respectively. In all scenarios and for all performance measures, the results for our backward selection procedure with the *p*.05 variable relevance criterion were very similar to the results when using the GKS variable relevance criterion. To simplify figures, we have shown results only for the simpler *p*.05 version of backward selection.

**Fig. 2:**
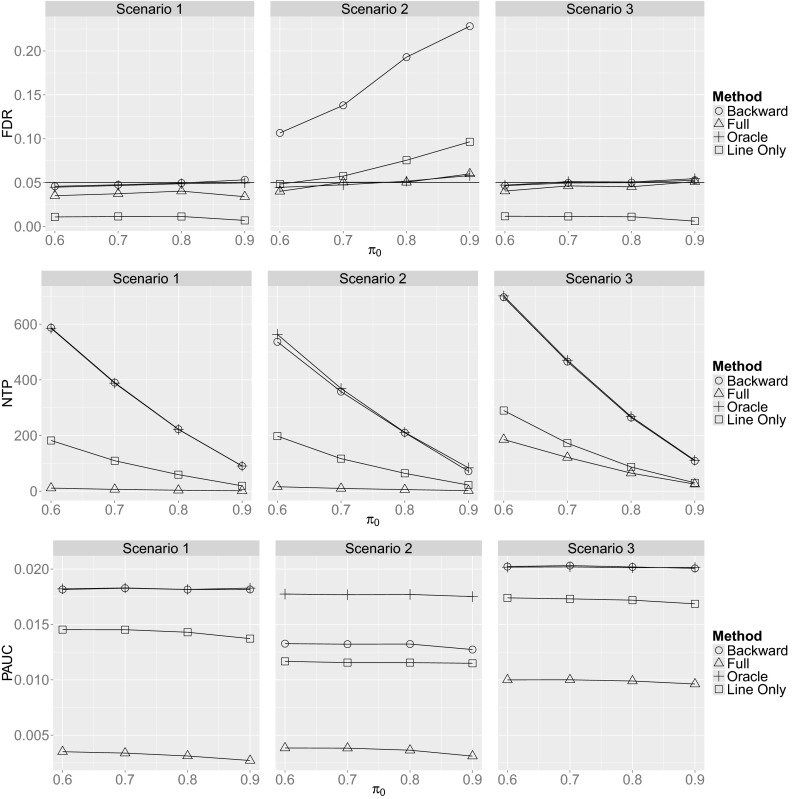
Empirical estimates of incurred false discovery rate (FDR), the average number of true positive (NTP) detections of differential expression, and the average partial area under the receiver operating characteristic curve (PAUC) from 100 replicates as a function of $\pi _0 \in \{0.6, 0.7, 0.8, 0.9\}$ for Backward, Full, Line Only, and Oracle methods and all three simulation scenarios. Standard errors of means (not shown to improve clarity of plots) were no larger than 0.0125, 4.6, and 0.00017 for FDR, NTP, and PAUC, respectively.

A summary of the results for simulation scenario 1 is displayed in the left column of Fig. 2. All methods provided approximate control of the FDR at or below 5 %. The Full approach was slightly conservative while the Line Only approach was very conservative, with actual FDR around 1 %. In terms of power for detecting DE genes and the ability to distinguish EE genes from DE genes, Backward performed as well as Oracle, while the Full and Line Only procedures exhibited far lower NTP and PAUC on average. The backward selection procedure was able to match the Oracle procedure in this scenario because the correct set of relevant covariates was chosen by backward selection ($\hat{\mathcal {S}}^*={\mathcal {S}}^*$) for around 80 % of the datasets. When backward selection failed to identify the exact set of relevant covariates ($\hat{\mathcal {S}}^*\ne {\mathcal {S}}^*$), the selected set was typically a small superset of the true set ($\hat{\mathcal {S}}^*\supset {\mathcal {S}}^*$) so that the fitted model was correct, though slightly more complicated than necessary due to the inclusion of one or (rarely) more irrelevant covariates.

The results for simulation scenario 2 are summarized in the second column of Fig. 2. Backward selection matched the Oracle procedure with respect to power (as measured by average NTP) and outperformed all methods except Oracle with respect to PAUC. Backward selection, however, failed to control FDR at 5 % for all values of $\pi _0 \in \{0.6,0.7,0.8,0.9\}$. The incurred FDR rate was more than four times the nominal level when $\pi _0=0.9$. The Line Only method also failed to control FDR for $\pi _0=0.8$ and 0.9, but the departures from the target 5 % rate were not as severe for Line Only as for Backward.

**Table 2:** The average number of false discoveries over 100 replicates as a function of $\pi _0$ and the true covariate set used to generate data in simulation scenario 2.

$\pi _0$	${\mathcal {S}}_6$	${\mathcal {S}}_7$	${\mathcal {S}}_8$	${\mathcal {S}}_9$	Total
0.6	46.57	5.47	5.62	6.44	64.10
0.7	44.40	4.14	3.99	4.85	57.3
0.8	41.44	2.77	2.64	3.45	50.30
0.9	23.58	1.17	1.43	1.44	27.62

The failure of the backward method to control FDR can be explained as follows. In simulation scenario 2, the true set of covariates is ${\mathcal {S}}_6$ for 1250 genes, ${\mathcal {S}}_7$ for 1250 genes, ${\mathcal {S}}_8$ for 1250 genes, and ${\mathcal {S}}_9$ for 1250 genes. Despite different sets of relevant covariates for different genes, the backward procedure, by design, selects one common set of covariates for all genes for reasons explained in Sect. 1. Backward selections chose ${\mathcal {S}}_7$ for more than 90 of the 100 datasets on average across $\pi _0\in \{0.6,0.7,0.8,0.9\}$. Because ${\mathcal {S}}_6 \supset {\mathcal {S}}_7 \supset {\mathcal {S}}_8 \supset {\mathcal {S}}_9$, selecting ${\mathcal {S}}_6$ would guarantee that all relevant covariates were included in the model for each gene. However, ${\mathcal {S}}_6$ includes *RFI*, which is strongly associated with *Line* as discussed in Sect. 4.3. The lack of orthogonality between the *Line* indicator variable and *RFI* reduces the significance of the *Line* partial regression coefficient in models that include both *Line* and *RFI*. Decreased significance of the *Line* partial regression coefficient reduces the number of *Lineq*-values less than or equal to 0.05 and discourages selection of ${\mathcal {S}}_6$ by our backward selection procedure. For EE genes whose true covariate set is ${\mathcal {S}}_6$, the partial regression coefficients for *RFI* and *Line* are nonzero and zero, respectively. However, when models excluding *RFI* are selected (e.g., ${\mathcal {S}}_7$) and fit to the data, the association between gene expression and *RFI* and between *RFI* and *Line* leads to a nonzero partial regression coefficient for *Line* in the fitted model. In the notation of Sect. 2, we have $\varvec{\beta }_{g1|{\mathcal {S}}_6}=\varvec{0}$ and $\varvec{\beta }_{g1|{\mathcal {S}}_7}\ne \varvec{0}$ for EE genes whose true covariate set is ${\mathcal {S}}_6$. When the selected covariate set is ${\mathcal {S}}_7$ for such genes, the null hypothesis $H_{0g1}^{{\mathcal {S}}_7}:\varvec{\beta }_{g1|{\mathcal {S}}_7}= \varvec{0}$ is correctly rejected, but this leads to a false discovery of differential expression in our simulation set up because $\varvec{\beta }_{g1|{\mathcal {S}}_6}=\varvec{0}$. Table 2 confirms that the vast majority of false discoveries by the Backward procedure occurred for genes whose relevant covariate set is ${\mathcal {S}}_6$.

Results for simulation scenario 3 are presented in the third column of Fig. 2. Recall that scenario 3 is identical to scenario 2 except that the strong association between the *RFI* covariate and the *Line* factor was eliminated by centering *RFI* values on zero within each line by subtracting within-line *RFI* averages. The resulting orthogonality between *RFI* and *Line* improved the performance of all methods with respect to all performance criteria when compared to both simulation scenarios 1 and 2. Backward performed as well as Oracle even though the relevant set of covariates differed from gene to gene. For approximately 75 % of the datasets, covariate set ${\mathcal {S}}_6$ was selected so that fitted models included the relevant covariates, along with 0, 1, 2, or 3 extra covariates depending on the gene. The loss of denominator degrees of freedom for including up to three irrelevant covariates was negligible in this case. However, the loss in power was substantial when all covariates were used, as shown by the relatively poor performance of the Full method.

## Discussion

The proposed backward selection algorithm provides a practical method for identifying and controlling for the effects of relevant covariates in the analysis of RNA-seq data. In the past, we have used visual inspection of *p*-value histograms (like those in Fig. 1) to identify and remove irrelevant covariates from models for RNA-seq data. The proposed backward selection algorithm provides a well-defined formalization of this process. This section discusses limitations, variations, and extensions of the backward selection algorithm.

### Combining Model Selection and Inference

Caution is in order any time the same dataset is used both to select a model and to perform statistical inference with the selected model (see Miller 2002, for example). We may avoid some problems associated with double use of data because of an important difference between the work we have presented here and traditional work on model selection and inference. While most past work focuses on a single response variable, we combine information from thousands of response variables when choosing the common set of variables to include in the model for each response. Although our backward selection algorithm uses data from all genes, excluding the data from any one gene would be very unlikely to change the set of selected covariates. Thus, we can view the model used to make inferences about any single gene as being selected using data from other genes. This separation between the data used for model selection and data used for inference could be partly responsible for the good inferential performance following backward selection exhibited in the simulation results of Sect. 4.4.

### Backward Selection with Other RNA-seq Analysis Methods

The reasoning behind our backward selection algorithm rests on the claim that including irrelevant covariates and excluding relevant covariates in models for gene expression analysis results in power loss for scientific discovery. Support for this claim is given in Sect. 2.2 and in the simulation results of Sect. 4.4. Our argument depends to some extent on the quasi-likelihood approach implemented in QuasiSeq and does not directly apply to other inference methods that do not account for lack of model fit with quasi-dispersion parameter estimates and do not account for model complexity with denominator degrees of freedom. Thus, further study is required before our backward selection algorithm could be recommended for use with other RNA-seq analysis packages. However, of the many methods available for RNA-seq analysis other than QuasiSeq, one approach does stand out as a good candidate for use with backward selection. The voom approach (Law et al. 2014) in conjunction with the R package limma (Ritchie et al. 2015) involves weighted linear model analysis of log-transformed RNA-seq read counts. The limma estimates of linear model error variances are analogous to the quasi-dispersion estimates of QuasiSeq, and both methods of inference involve *F* statistics whose denominator degrees of freedom are derived from the same basic argument. For these reasons, we expect the proposed backward selection to work well with voom/limma analysis.

### Measures of Covariate Relevance

We have suggested two related measures of covariate relevance to use in backward selection. We have found that both measures perform very similarly across the simulation scenarios we considered. The *p*.05 criterion has an advantage of simplicity but could be criticized because of the somewhat arbitrary 0.05 *p*-value threshold. Alternative thresholds could be considered, but we do not expect much variation in performance across thresholds near 0.05 because of the similar performance of *p*.05 and GKS, which is threshold free. Both the *p*.05 and GKS criteria provide reasonable ways to detect departures from uniformity toward distributions stochastically smaller than uniform, and both criteria produce similar sequences of models that permit effective model selection.

### Direct Versus Indirect Associations and Automatic Covariate Inclusion

For model selection, we have proposed choosing the model (from those in the backward selection sequence) that maximizes the number of declarations of differential expression subject to control of FDR at a desired nominal level. Despite the greedy nature of this selection criterion, we found that the approach worked well except for challenging genes where there is no direct association between gene expression and the primary factor of interest, but rather only indirect association that results from strong association between the primary factor of interest and a covariate that is directly associated with gene expression. In this situation, illustrated with simulation scenario 2 in Sect. 4.2, the proposed backward selection procedure failed to control FDR. However, most false discoveries in this case were not incorrect conclusions if the declarations of differential expression are stated as associations between gene expression and the primary factor of interest while controlling for the effects of the selected covariates. In our analysis of the actual RFI RNA-seq dataset in Sect. 3, we must be careful to acknowledge that some of the gene expression levels declared to be significantly associated with *Line* may be indirectly associated with *Line* and only directly associated with *RFI* or other covariates that were not included in the selected set.

In the RFI application, we are fortunate that either direct or indirect associations between expression and *Line* are of interest. In other applications where researchers are specifically interested in distinguishing direct effects of a primary factor of interest from indirect effects due to a covariate, such covariates should be automatically included in the model. More generally, if scientific questions of interest dictate that one or more covariates be included in the model, the fate of such covariates should not be decided by backward selection; rather, such covariates should be part of every model considered, just as the intercept term was, by default, part of every model we fit to the RFI dataset. Similarly, covariates that indicate restrictions on randomization in the experimental design (like *Block* in the RFI example) may be included in the model by default rather than subjected to backward selection. The backward selection algorithm’s primary purpose is to identify and account for covariates that are not part of the model a priori but are relevant in the sense that they explain non-negligible residual variation in transcript abundance levels beyond that explained by factors that are part of the model a priori. Accounting for such relevant covariates can boost power for discovery of differential expression that is of primary scientific interest.

### Backward Selection to Account for Unobserved Covariates

In contrast to our paper, which has focused on adjusting for the effects of observed covariates, Leek and Storey (2007) and Leek (2014) have proposed surrogate variable analysis (SVA) as a strategy for dealing with unobserved covariates in differential expression analysis of microarray data and RNA-Seq data, respectively. It is possible to combine SVA with our backward selection strategy to simultaneously account for both observed and unobserved covariates in RNA-seq analysis. Using our RFI RNA-seq dataset as an example, we applied the approach of Leek (2014) as implemented in the svaR package available on Bioconductor (Gentleman et al. 2004). After accounting for the effects of all 14 observed variables in our dataset, SVA detected one unobserved covariate and estimated values for a surrogate variable to be used in place of the unobserved covariate. We then applied our backward selection algorithm as described in Sect. 3, except that we included the surrogate variable among our other covariates. The surrogate variable was removed from the model on the fourth iteration, and the final selected model was identical to the model chosen in Sect. 3. Although considering unobserved covariates turned out to be irrelevant for the analysis of our example dataset, accounting for such variables may be crucial in other cases.

### Backward Selection When Multiple Factors are of Interest

We have described our method for the important special case where a single categorical factor is of primary scientific interest. The backward selection algorithm can be trivially extended to handle cases where a single quantitative variable is of primary interest. If multiple factors (quantitative, categorical, or a combinations of the two) are of interest, there are multiple variations of the algorithm that could be considered. We will highlight two options by focusing on the case where two factors (say *A* and *B*) are of interest.

First, suppose the part of the model involving the factors of interest is specified a priori so that backward selection will focus only on eliminating irrelevant covariates from the model. For example, suppose we will include $A,\, B$, and $A \times B$ interaction effects in our model regardless of what the data imply about the significance of these effects. To choose what covariates to include in a model with $A,\,B$, and $A \times B$ interaction effects, we could apply our backward selection algorithm as before by treating the $A,\, B$, and $A \times B$ interaction effects as the effects associated with a single factor of primary interest. In the notation of Sect. 2, we would define $\varvec{x}_{i1}$ and $\varvec{\beta }_{g1|{\mathcal {S}}}$ so that $\varvec{x}_{i1}'\varvec{\beta }_{g1|{\mathcal {S}}}$ represents the sum of the appropriate $A,\, B$, and $A \times B$ partial regression coefficients for unit *i* in the model with variables indicated by ${\mathcal {S}}$. Backward selection could then proceed exactly as defined in Sect. 2.3. The joint significance of the $A,\, B$, and $A \times B$ partial regression coefficients would determine when to stop backward selection and which model in the backward selection sequence to choose.

Now suppose the part of the model involving the factors of interest is not fully specified a priori but instead will be chosen based on an examination of the data. For example, suppose the researchers are interested in the main effects of factors *A* and *B* but do not want to study $A \times B$ interaction effects unless the data indicate that these effects are important. One strategy is to treat the $A \times B$ interaction effects as we would the effects of any other categorical covariate. Without loss of generality, interaction effects for unit *i* could be coded in $\varvec{x}_{i2}$ and other covariates specified by $\varvec{x}_{i3},\ldots ,\varvec{x}_{ik}$. Additive effects for *A* and *B* and unit *i* would be coded in $\varvec{x}_{i1}$, and joint significance of the partial regression coefficients on $\varvec{x}_{i1}$ would be used to stop backward selection and choose the model. If the selected model includes $A \times B$ interaction effects, subsequent inferences would be made for each gene using a model that includes $A,\, B$, and $A \times B$ interaction effects, along with any other selected covariates. If interaction effects are removed by the backward selection algorithm, then subsequent inferences for each gene could focus on *A* and *B* main effects while accounting for the effects of other relevant covariates without further consideration of $A \times B$ interactions.

Even in the relatively simple two-factor scenario described above, there are other strategies worth considering that we have not described here. Determining the relative merits of various strategies requires a more formal problem statement, including a clear description of the tests to be conducted after model selection, the desired error control properties for each set of tests, and priorities for discovery of the multiple types of differential expression that arise when multiple factors are of scientific interest. Such details are beyond the scope of the current article but worth considering in future research.

## Appendix: Description of Variables in the RFI Dataset

- $\varvec{x}_{\cdot 1}= {\mathbf {Line}}$ is the categorical factor of primary scientific interest. Line has two levels, which correspond to the HRFI and LRFI selection lines. Among the 31 pigs in this study, 15 were from the LRFI line and 16 were from the HRFI line.
- $\varvec{x}_{\cdot 2}= {\mathbf {RFI}}$ is a continuous covariate that provides a measure of the residual feed intake for each of the 31 pigs from which blood samples were drawn for RNA-seq analysis. Pigs in the HRFI line tend to have high *RFI* values, while pigs in the LRFI line tend to have low *RFI* values.
- $\varvec{x}_{\cdot 3}={\mathbf {Diet}}$ is a categorical factor with two levels corresponding to the two diets (high fiber, low energy vs. low fiber, high energy) that were fed to the pigs in this study. Approximately half the pigs within each line were fed each diet. Because RNA-seq analysis was performed on blood samples collected prior to the initiation of the two diets, this factor is not expected to be associated with the transcript abundance levels measured by RNA-seq. However, other covariates in this study may be associated with *Diet*. For example, feed consumption on the assigned diet played a role in the calculation of *RFI* values, and pigs on the high fiber, low energy diet tended to have higher *RFI* values than pigs on the low fiber, high energy diet.
- $\varvec{x}_{\cdot 4}= {\mathbf {Baso}}$ is a continuous covariate that provides a measure of the concentration of basophil cells in the blood sample drawn from each pig.
- $\varvec{x}_{\cdot 5}= {\mathbf {Eosi}}$ is a continuous covariate that provides a measure of the concentration of eosinophil cells in the blood sample drawn from each pig.
- $\varvec{x}_{\cdot 6}= {\mathbf {Lymp}}$ is a continuous covariate that provides a measure of the concentration of lymphocyte cells in the blood sample drawn from each pig.
- $\varvec{x}_{\cdot 7}= {\mathbf {Mono}}$ is a continuous covariate that provides a measure of the concentration of monocyte cells in the blood sample drawn from each pig.
- $\varvec{x}_{\cdot 8}= {\mathbf {Neut}}$ is a continuous covariate that provides a measure of the concentration of neutrophil cells in the blood sample drawn from each pig.
- $\varvec{x}_{\cdot 9}= {\mathbf {Concb}}$ is a continuous measure of the RNA concentration in each sample before globin depletion (a step that is necessary to focus sequencing efforts on messenger RNA molecules other than highly abundant globin messenger RNA in each blood sample).
- $\varvec{x}_{\cdot 10}= {\mathbf {Conca}}$ is a continuous measure of the RNA concentration in each sample after globin depletion.
- $\varvec{x}_{\cdot 11}={\mathbf {RINb}}$ is a continuous measure of RNA integrity within each sample before globin depletion.
- $\varvec{x}_{\cdot 12}={\mathbf {RINa}}$ is a continuous measure of RNA integrity within each sample after globin depletion.
- $\varvec{x}_{\cdot 13}= {\mathbf {Block}}$ is a categorical factor with four levels corresponding to the four blocks used to organize sample collection and processing. Initially, each block involved eight samples, two for each combination of *Line* and *Diet*. One LRFI sample from the first block was removed from the study due to low-quality RNA.
- $\varvec{x}_{\cdot 14}= {\mathbf {Order}}$ is a categorical factor with eight levels indicating the random order samples were processed within each block.
